# Semiology and neurophysiology of clonic seizures in children: a retrospective study

**DOI:** 10.3389/fneur.2025.1597943

**Published:** 2025-08-14

**Authors:** Qiao Hu, Yuanyuan Luo, Tingsong Li, Siqi Hong, Ping Yuan, Li Jiang

**Affiliations:** ^1^Department of Neurology, Children’s Hospital of Chongqing Medical University, National Clinical Research Center for Child Health and Disorders, Ministry of Education Key Laboratory of Child Development and Disorders, China International Science and Technology Cooperation Base of Child Development and Critical Disorders, Chongqing Key Laboratory of Child Neurodevelopment and Cognitive Disorders, Chongqing, China; ^2^Department of Rehabilitation, Children’s Hospital of Chongqing Medical University, Chongqing, China

**Keywords:** clonic seizure, semiology, neurophysiology, children, compare with adults

## Abstract

**Objectives:**

To identify and quantify clonic seizures in children, we retrospectively reviewed the clinical symptoms and neurophysiology of them.

**Methods:**

Data were obtained from 24 patients presenting with 34 clonic seizures, and their video-electroencephalography (EEG) recordings were examined for symptomatology and ictal EEG characteristics. Additionally, synchronous electromyography (EMG) data from 17 patients were analyzed.

**Results:**

Our quantitative analysis demonstrated high diagnostic precision in lateralizing focal clonic seizures, with 90.9% exhibiting contralateral hemispheric concordance. The perirolandic region emerged as the predominant seizure-onset zone (62.85%), while paroxysmal rhythmic monomorphic activity constituted the most frequent ictal EEG pattern (72.72%). Semiological evaluation revealed preferential lower limb involvement at onset (38.23% of seizures) followed by upper limb manifestations (14.7%), with propagation patterns dominated by medial-to-lateral spread (63.63%) over purely lateral dissemination (36.36%). Neurophysiological profiling identified a mean EEG–EMG discharge latency of 115.88 msec. Notably, epilepsy represented the primary underlying etiology (83.83%), distinguishing pediatric clonic seizures from adult populations where structural lesions predominate.

**Conclusion:**

The lateralizing value, seizure-onset zone and EEG seizure pattern in childhood clonic seizures exhibited consistency with those observed in adults. Nonetheless, distinctions were noted in the initial affected body parts, latency, and etiology compared to adult cases. The delineated characteristics in this study could facilitate the recognition and assessment of clonic seizures during video-EEG monitoring in children.

## Introduction

A seizure is defined as “a transient occurrence of signs and/or symptoms due to abnormal excessive or synchronous neuronal activity in the brain (1).” Clonic seizures are defined as symmetric or asymmetric jerking movements that are regularly repetitive and involve the same muscle groups (2). Myoclonic seizures are defined as sudden, brief (<100 msec), involuntary single or multiple contraction(s) of muscles(s) (2). Although clonic seizures are usually thought of as repetitive rhythmic myoclonic seizures, these seizures are not easily understood.

Clonic seizures were first described by Louis in 1827 (3). In 1870, Jackson further expanded on this type of attack (3). Although some studies in the literature have analyzed the lateralization value of focal clonic epilepsy, few studies have systematically analyzed the clinical semiology and neurophysiology of clonic seizures (4–8). Fotedar reported that in a cohort with an average age of 48.8 years, clonic seizures propagated from the lower face to the upper face and from the distal hand to the proximal arm (8). However, the manifestations of seizures are age specific and depend on the maturation of the brain (1). Therefore, we systematically summarized the clinical semiology and neurophysiology of clonic seizures in children from our center to gain a more in-depth understanding of these seizures.

## Methods

We retrospectively reviewed the electroencephalography (EEG) data of children who underwent video-EEG monitoring during clonic seizures (unilateral or bilateral) at the Children’s Hospital of our Medical University between 2020 and 2024. All included children had clear video records of clonic seizures. Any videos in which the affected body part was partially or completely hidden were excluded. We also excluded children with generalized tonic–clonic (GTC) seizures and those with poor-quality EEG during the ictal period.

On the basis of these criteria, we identified 24 patients (n1) with 34 seizures (n2). More than 1 seizure per patient were included only if the initially affected body part or the propagation pattern of the clonic seizure differed from the first one. Different propagation pattern refers to the different main manifestations of the ictal period and the different order of occurrence.

Twelve of the children were males, and 12 were females. The age of the children ranged from 2 months to 6 years (mean, 3.45 years). This cohort included patients with chronic epilepsy and acute symptomatic seizures.

Video-EEG was performed using an EEG-1200 system from Nihon Kohden (Tokyo, Japan). The EEG electrodes were placed according to the standard 10–20 international system. In 17 patients (21 seizures), clearly analyzable surface electromyography (sEMG) was also performed, with the electrodes placed bilaterally on the deltoid and quadriceps muscles. Two neurophysiologists analyzed all video-EEG and sEMG data. This study was approved by the Ethics Review Committee of the Children’s Hospital of Chongqing Medical University. Consent to participate Informed consent was obtained from the children’s parents or legal guardians.

### Seizure semiology: EEG and video analyzes

We analyzed the following characteristics:

1. Initial body parts involved in the clonic seizure.
2. Propagation pattern.
3. Semiology preceding and following the seizure.
4. Seizure-onset zone, EEG seizure pattern, and lateralizing value.

### Neurophysiology: sEMG analysis

We analyzed the following characteristics:

1. Rhythmicity of EMG bursts (rhythmic means EMG with clear evolution in amplitude and duration, while arrhythmic means with no evolution).
2. Duration of EMG bursts.
3. EEG-to-sEMG latency.

### Statistical analysis

The statistical software package SPSS 25.0 was used for all analyzes. The data are expressed as the means ± standard deviations and medians. Data from this children study are descriptive only. Because adult patients were relying only on data from previous studies, thus no further statistically compared.

## Results

### Seizure semiology: EEG and video analyzes

#### Seizure-onset zone, EEG seizure pattern, and lateralizing value

**Figure 1:**
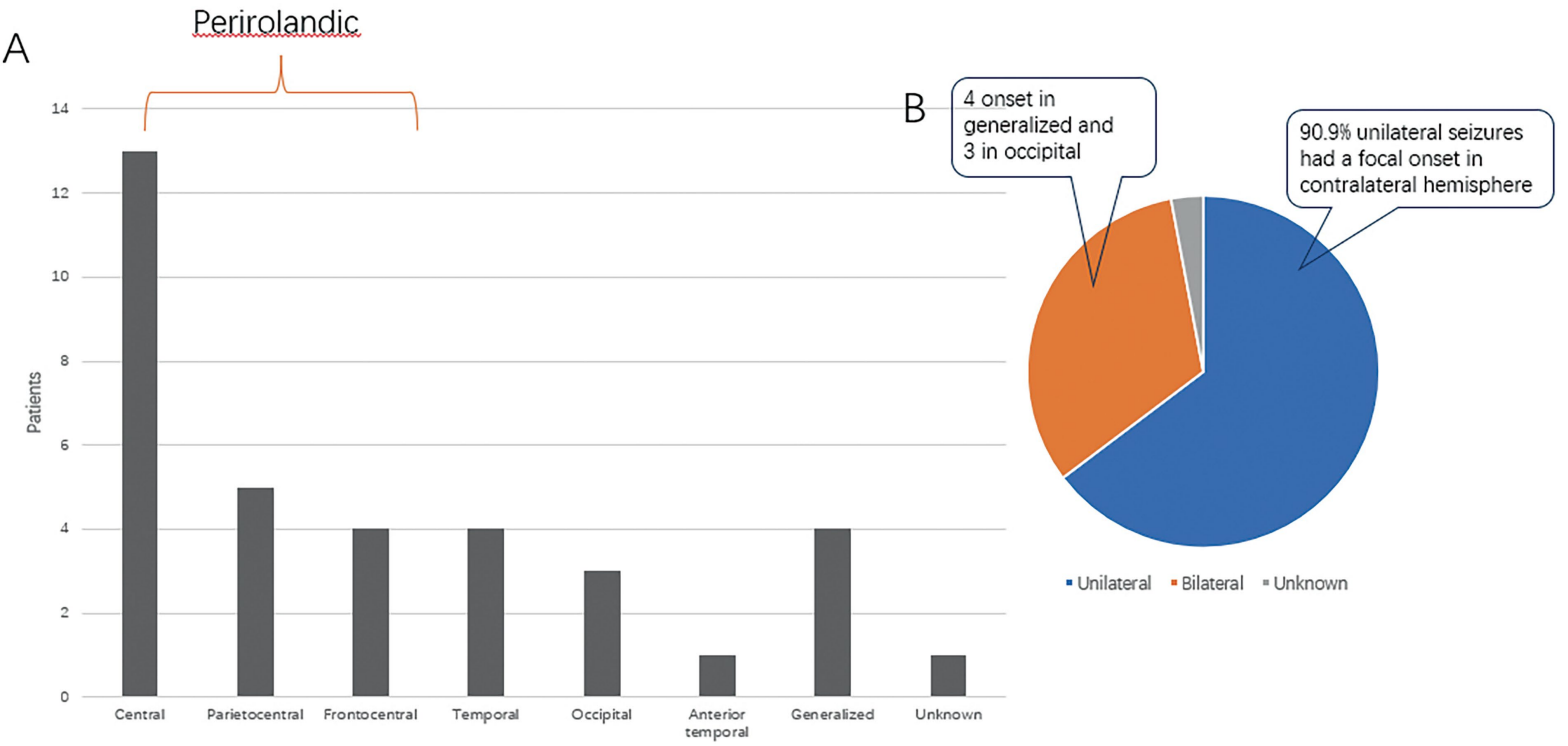
Seizure-onset zones and lateralizing values. **(A)** Graph showing seizure-onset zones. **(B)** Pie chart showing the distribution of unilateral and bilateral clonic seizures.

Figure 1A shows the distribution of seizure-onset regions in our cohort. Overall, the pericentral regions (Parietocentral, Frontocentral and central) were the most common seizure-onset regions (62.85%).

Among the 34 seizures, 22 had unilateral (focal) clonus. Of those 22 seizures, 90.9% (20/22) of the clonic seizures occurred on the contralateral side. There were 11 cases of bilateral clonus, 4 of which had generalized onset and 3 of which were from the occipital region (Figure 1B).

**Figure 2:**
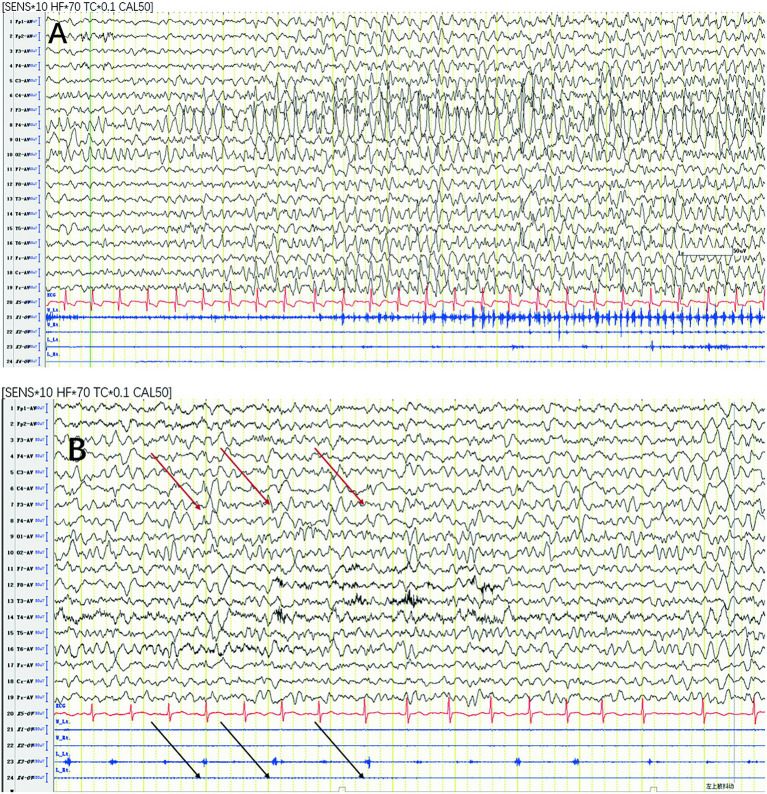
Most common seizure patterns. **(A)** Paroxysmal rhythmic monomorphic theta-delta activity. **(B)** Periodic epileptiform discharges with synchronized EMG bursts of the left deltoid (black arrows) time locked to the right central LPDs (red arrows). LPD, lateralized periodic discharge.

Paroxysmal rhythmic monomorphic theta-delta activity (72.72%) (Figure 2A) and periodic epileptiform discharges (15.15%) (Figure 2B) were the most common seizure patterns. The remaining 4 cases were characterized by a spike–wave pattern (12.12%) and had generalized onset.

For all focal-onset seizures, the proportion of seizures characterized by periodic epileptiform discharges was greater in the perirolandic group than that of focal-onset seizures in different onset zones (68.18% vs. 41.66%). The most common EEG seizure pattern in generalized-onset seizures was a spike–wave pattern (75%).

#### Initial body part involved in clonic seizures

**Figure 3:**
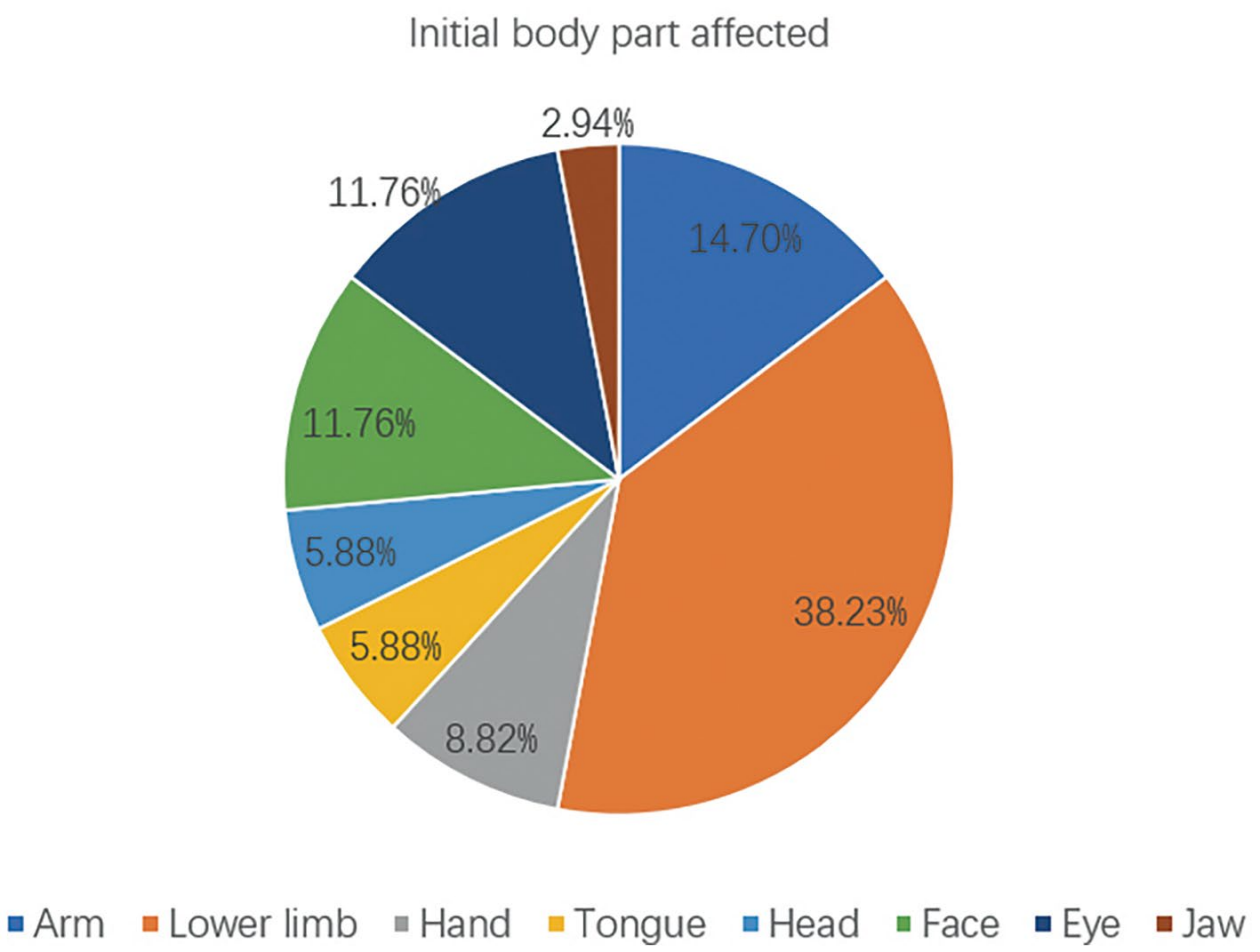
Pie chart distribution of the most common initial affected body parts in clonic seizures.

Among the 34 seizures, the initial affected body part was the lower limb in 13 (38.23%) seizures, followed by the arm in 5 (14.7%) seizures and the face and eye in 4 (11.76%) seizures (Figure 3). Overall, the extremities and face (arm+lower limb+face+eye) were the most common initial affected body parts (64.69%). These findings are consistent with the substantial representation of the upper/lower extremities and face in the motor homunculus.

#### Propagation patterns

According to the propagation paths, we classified the propagation modes into the following types:

1. Anatomical medial-to-lateral spread (lower limb→arm).
2. Anatomical lateral spread (tongue→jaw→face→arm).
3. Anatomical lateral-to-medial spread (arm→lower limb; hand→arm→lower limb).

**Figure 4:**
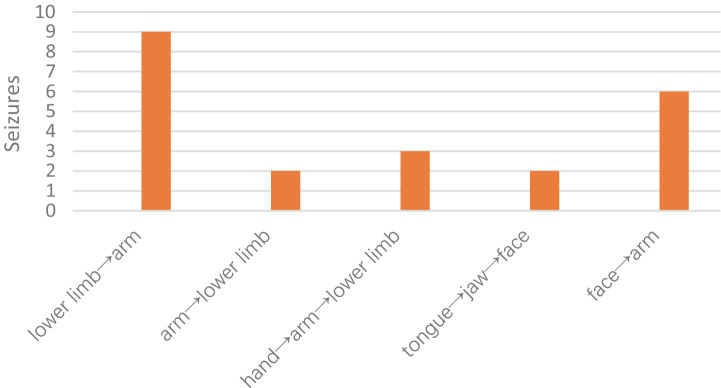
Graph of the various propagation patterns.

Overall, the most common pattern was the medial-to-lateral spread pattern (63.63%), followed by the lateral spread pattern (36.36%). The lower extremities were the most commonly affected muscle group in all modes of propagation (63.63%) (Figure 4).

### Symptoms preceding and following seizures

The symptoms preceding clonic seizures were numbness in 1 patient, akinesia in 1 patient, tonic movements in 2 patients, and deflection in 2 patients. No symptoms following seizures were reported or observed.

### Neurophysiology: sEMG analysis

In total, 21 clonic seizures were monitored using sEMG electrodes. On the basis of the rhythmicity of the EMG bursts, we classified the clonic seizures into arrhythmic and rhythmic. The most common EEG seizure pattern associated with both arrhythmic and rhythmic clonic seizures was paroxysmal rhythmic monomorphic activity (88.88 and 100%, respectively).

#### Arrhythmic clonic seizures (*n* = 11)

The EMG bursts were synchronous with no evolution in amplitude or duration. The mean duration of EMG bursts was 123.89 msec. The mean latency from the onset of EEG discharge to the onset of an EMG burst was 115.88 msec. The mean latency from the peak of EEG discharge to the onset of an EMG burst was 57.38 msec.

#### Rhythmic clonic seizures (*n* = 10)

The EMG bursts were synchronous, similar to arrhythmic clonic seizures, with clear evolution in amplitude and duration from the first half of the seizure to the second half. The average duration of EMG bursts increased from 64.92 to 260.08 msec, from the first half of the seizure to the second half. Similarly, the average EMG burst amplitude increased by approximately 198%.

### Etiology

The etiologies and other various characteristics of clonic seizures are summarized in Table 1. Epilepsy was the most common etiology in our study.

**Table 1:** Various characteristics of clonic seizures in our study.

Age(y)	Sex		Etiology		Onset regions		Seizure pattern		Initial body part involved		Propagation patterns	
3.45 ± 2.62	Male	12	Epilepsy	20	Central	13	Theta-delta activity	25	Lower limb	13	Medial-to-lateral spread pattern	14
	Female	12	Structural	3	Parietocentral	5	Periodic epileptiform discharges	5	Arm	5	Lateral spread pattern	8
			Genetic	3	Frontocentral	4	Spike–wave pattern	3	Hand	3		
			SeLECTS	1	Temporal	4	Unknown	1	Tongue	2		
			LGS	2	Occipital	3			Head	2		
			West Syndrome	1	Anterior temporal	1			Face	4		
			Epilepsy	10	Generalized	4			Eye	4		
			Encephalitis	2	Unknown	1			Jaw	1		
			Tumors	1								
			Rasmussen syndrome	1								

## Discussion

Few articles have studied the localization and lateralization value of clonic seizures, and those that have were not detailed (4–7, 9, 10). Only one paper reported simultaneous EEG and sEMG recordings (8). The average age of patients in that study was 48.8 years, and cerebrovascular causes and brain tumors were the most common etiologies (8). Given the obvious differences between children and adults, we collected a cohort of children with an average age of 3.45 years and epilepsy as the most common cause; the clinical semiology, seizure-onset zone, lateralizing value, and neurophysiology of clonic seizures were characterized in this pediatric cohort and compared with those of the adult cohort. To our knowledge, this study will be the first study of the semiology and neurophysiology of clonic seizures in a pediatric cohort.

### Clinical semiology

Our cohort study revealed that the lower limbs were the most common initially affected body parts, whereas the arms and hands were most common in the adult cohort (8). However, it is possible that the prevalence of lower limb onset may be due to the relatively poor expression and cognitive abilities of children, and clonus movements of lower limbs are more likely to attract the attention of guardians than those of the upper limbs. Rhythmic clonus in the lower limbs can cause children to become unstable or even fall down.

In both our cohort and adults, the most common seizure-onset zone for focal clonic seizures was the perirolandic region (8). Since the symptomatic area of clonic seizures is believed to be the primary motor cortex of the precentral gyrus, all of these findings are consistent with the large representation of the upper/lower extremities and face in the motor homunculus (11, 12).

The initial descriptions of clonic seizures were made by Sir John Hughlings Jackson; later, Penfield and Jasper found that epileptic discharges can propagate up or down the precentral gyrus, resulting in the movement of the seizures from body part to another (13). On the basis of these propagation paths, we classified the propagation modes into three types, and the most common pattern was the medial-to-lateral spread pattern (lower limb→arm), followed by lateral spread (tongue→jaw→face→arm). The lateral spread pattern (hand to arm/shoulder or lower face to upper face) was the most common propagation mode in adults (8). However, the propagation of seizure activity in children and adults is not simply due to cortical contiguity. For example, clonic seizures spread from the face to the arm rather than to the hand and then to the arm, even though the face and hand regions are adjacent. Propagation may also be dependent on the functional connectivity of cortical areas. Using functional magnetic resonance imaging (fMRI), Evan M. Gordon found that the classic homunculus is interrupted by regions with distinct connectivity (14). This finding explains the complex propagation pattern of clonus.

### Lateralizing value

In our study and a previous study in adults, the lateralizing value of focal clonic seizures to the contralateral hemisphere was 90.9–100% (8). To our knowledge, this study is the largest study of clonus in a pediatric cohort with simultaneous video-EEG analysis. In the study by Gallmetzer et al., 29 adult patients with 40 focal clonic seizures exhibited contralateral laterality in 35 seizures with ictal EEG data (6). Other studies in the literature have analyzed the laterality values in clonic seizures with a small number of patients. In general, the values ranged from 81.3 to 100%, which is consistent with our study (4–7, 10, 15).

### Neurophysiology of clonic seizures

The most common seizure-onset zone for focal clonic seizures was the perirolandic region in both children and adults. This finding is consistent with previous studies showing that the most likely pathogenic zone of clonic seizures is the primary motor area in the precentral gyrus (9, 12). The epileptic discharges based in the perirolandic region activate pyramidal tract neurons, which produce the brief muscle contractions observed on sEMG that are time locked to epileptic discharges (9, 12). This hypothesis is supported by our research.

Clonic seizures have unique sEMG characteristics that are similar to the clonic responses obtained by electrical stimulation of the primary motor area. Electrical stimulation studies have shown that low-frequency and high-frequency stimulation of the primary motor area can produce different clonic responses. Low-frequency (<20 Hz) electrical stimulation produces simple EMG bursts (≤50 msec) composed of single motor unit potentials associated with periodic epileptiform discharges. High-frequency (≥20 Hz) stimulation produces complex EMG bursts (>50 msec) composed of multiple motor unit potentials associated with paroxysmal rhythmic monomorphic theta-delta activity (8, 12). In our study, the mean duration of EMG bursts in patients with clonic seizures was longer than 50 msec because paroxysmal rhythmic monomorphic theta-delta activity (72.72%) was the most common seizure pattern. This finding is consistent with studies in adult cohorts (8).

sEMG characteristics can aid in the diagnosis and long-term monitoring of clonic seizures (16, 17). Thus, according to the rhythmicity of the EMG bursts, we classified the clonic seizures into arrhythmic and rhythmic. Rhythmic and arrhythmic clonic seizures in adult and pediatric cohorts are compared in Table 2. The latency from the onset or peak of EEG discharge to the onset of the EMG burst in arrhythmic clonic seizures is significantly longer in children than in adults. This difference may be related to the immaturity of neural networks and their easier generalization in children.

**Table 2:** Rhythmic and arrhythmic clonic seizures in adults and children.

	Rhythmic clonic seizures		Arrhythmic clonic seizures	
	Children	Adults	Children	Adults
Duration of EMG burst (msec)	64.92 to 260.08	188.5 to 320.6	123.89	154.5
EMG amplitude increase ratio	198%	150%	/	/
Latency from onset of EEG discharge to onset of EMG burst (msec)	/	/	115.88	55.5
Latency from peak of EEG discharge to onset of EMG burst (msec)	/	/	57.38	11.3

The observed prolonged EEG-to-EMG latency in pediatric populations has important implications for both seizure detection algorithms and presurgical planning. For automated seizure detection systems, this latency suggests the need for algorithm adjustments to account for delayed motor manifestations relative to electrographic onset, particularly since most current algorithms rely on temporal synchronization between EEG and EMG signals for optimal performance (18, 19). In presurgical evaluation, the latency differences may reflect altered neuronal propagation velocities within epileptogenic networks (20), which could serve as a potential biomarker for localizing the epileptogenic zone. Specifically, our findings align with studies showing that increased cortico-cortical evoked potential (CCEP) latencies correlate with the epileptogenic zone (20), suggesting that latency measurements could complement traditional localization methods. Furthermore, the interhemispheric latency differences our observed (21), may help distinguish lesional from nonlesional epilepsy cases during presurgical workup. These latency characteristics should be incorporated into multimodal presurgical evaluation protocols to improve surgical outcome predictions (21, 22). The findings also emphasize the need for pediatric-specific algorithm training datasets, as children demonstrate different neurophysiological patterns compared to adults (23, 24).

## Conclusion

In summary, in our pediatric cohort, the lateralizing value of focal clonic seizures to the contralateral hemisphere was 90.9%. The most common seizure-onset zone was the perirolandic region, and the most common EEG seizure pattern was paroxysmal rhythmic monomorphic activity. The onset zone, laterality and most common EEG patterns of clonic seizures in the pediatric cohort were highly consistent with those of adults, but the initial affected body parts (lower limbs/upper limbs) and propagation pattern (Medial-to-lateral spread pattern/ Face propagation) were different. The latency from the onset or peak of EEG discharge to the onset of an EMG burst in arrhythmic clonic seizures was significantly longer in children than in adults.

The characteristics described in this article may aid in the identification and quantification of clonic seizures during video-EEG monitoring in children. This study was a single-center retrospective study with a limited sample size and consent.

## Data Availability

The original contributions presented in the study are included in the article/supplementary material, further inquiries can be directed to the corresponding authors.
